# A Cell-based Screen in *Actinomyces oris* to Identify Sortase Inhibitors

**DOI:** 10.1038/s41598-020-65256-x

**Published:** 2020-05-22

**Authors:** Jason E. Gosschalk, Chungyu Chang, Christopher K. Sue, Sara D. Siegel, Chenggang Wu, Michele D. Kattke, Sung Wook Yi, Robert Damoiseaux, Michael E. Jung, Hung Ton-That, Robert T. Clubb

**Affiliations:** 1 Department of Chemistry and Biochemistry, University of California, Los Angeles, USA; 2 UCLA-DOE Institute of Genomics and Proteomics, University of California, Los Angeles, USA; 3 Division of Oral Biology and Medicine, University of California, Los Angeles, USA; 4 Department of Microbiology and Molecular Genetics, University of Texas Health Science Center, Houston, TX USA; 5 Department of Molecular and Medicinal Pharmacology, University of California, Los Angeles, USA; 6 California NanoSystems Institute, University of California, Los Angeles, USA; 7 Molecular Biology Institute, University of California, Los Angeles, 611 Charles Young Drive East, Los Angeles, CA 90095 USA

**Keywords:** High-throughput screening, Microbiology, Antimicrobials

## Abstract

Sortase enzymes are attractive antivirulence drug targets that attach virulence factors to the surface of *Staphylococcus aureus* and other medically significant bacterial pathogens. Prior efforts to discover a useful sortase inhibitor have relied upon an *in vitro* activity assay in which the enzyme is removed from its native site on the bacterial surface and truncated to improve solubility. To discover inhibitors that are effective in inactivating sortases *in vivo*, we developed and implemented a novel cell-based screen using *Actinomyces oris*, a key colonizer in the development of oral biofilms. *A*. *oris* is unique because it exhibits sortase-dependent growth in cell culture, providing a robust phenotype for high throughput screening (HTS). Three molecules representing two unique scaffolds were discovered by HTS and disrupt surface protein display in intact cells and inhibit enzyme activity *in vitro*. This represents the first HTS for sortase inhibitors that relies on the simple metric of cellular growth and suggests that *A*. *oris* may be a useful platform for discovery efforts targeting sortase.

## Introduction

Proteins displayed on the surface of bacterial pathogens play critical roles in the infection process by promoting bacterial adhesion to host tissues, acquisition of essential nutrients, evasion and suppression of the immune response and host-cell entry^1,2^. Gram-positive bacteria display virulence factors using sortases, cysteine transpeptidase enzymes that covalently attach proteins to peptidoglycan precursors or assemble pili^3–6^. Sortase enzymes are potential drug targets as they are required for the virulence of methicillin-resistant *Staphylococcus aureus* (MRSA), which causes a wide range of life-threatening diseases, such as pneumonia, meningitis, osteomyelitis, endocarditis, toxic shock syndrome, bacteremia, and sepsis^7^. These infections are major health concerns as they are estimated to cause 16,485 fatalities in the United States each year^8^. Sortase enzymes also contribute to the virulence of other clinically important pathogens, including among others: *Enterococcus faecalis*, *Listeria monocytogenes*, *Bacillus anthracis*, *Streptococcus pyogenes* and *Streptococcus pneumoniae*^9^. Thus, small-molecule inhibitors of sortase enzymes may prove to be useful antimicrobial agents to treat infections caused by MRSA and other bacterial pathogens.

The sortase enzyme in *S*. *aureus* (^Sa^SrtA) has been characterized to the greatest extent^3–6^. It recognizes protein substrates that harbor a C-terminal cell wall sorting signal (CWSS), which is comprised of a conserved LPXTG-type motif (where X denotes any amino acid), followed by a hydrophobic transmembrane segment and positively charged residues. The membrane-bound ^Sa^SrtA covalently attaches protein substrates to the peptidoglycan via a transpeptidation reaction by cleaving the LPXTG motif between the Gly and Thr residues and joining the cleaved LPXT to the cross-bridge peptide of lipid II, a peptidoglycan precursor. Catalysis occurs through a ping-pong mechanism that is initiated when the active-site cysteine nucleophile attacks the backbone carbonyl carbon of the threonine residue within the LPXTG motif, breaking the threonine-glycine peptide bond to create a thioacyl-linked sortase-protein complex^10–13^. The protein-lipid II product of the sortase catalyzed reaction is formed when the thioacyl substrate-enzyme intermediate is resolved by the amino group within lipid II. Cell wall synthesis reactions then incorporate the protein-lipid II product into the peptidoglycan, displaying the protein on the microbial surface. Over 3,100 species of bacteria contain genes encoding for sortases related to ^Sa^SrtA^14,15^, but in some instances these enzymes perform lysine-isopeptide transpeptidation reactions that construct pili virulence factors^16^.

Given its potential as a drug target, because sortase mutants are attenuated in virulence, considerable effort has been put forth to discover small molecule ^Sa^SrtA inhibitors^9,17–19^. Previously reported studies have searched for inhibitors by monitoring the activity of the purified ^Sa^SrtA enzyme *in vitro* using a Förster resonance energy transfer (FRET) assay. The FRET assay has been used to screen small-molecule compound libraries^20–23^ and to assess the potency of rationally designed peptidomimetics^24–27^, natural products^28–38^, and small molecules identified using virtual screening approaches^39–42^. While several inhibitors have been discovered, to the best of our knowledge, none have advanced into clinical trials. It is possible that some of these compounds are unable to effectively inhibit the enzyme in its natural context, the extra-cellular bacterial membrane where it may associate with components of the protein secretion and cell wall synthesis machinery. A cell-based assay for high-throughput screening (HTS) for sortase inhibitors could overcome this limitation, but has yet to be implemented in *S*. *aureus* because inhibiting ^Sa^SrtA activity does not significantly affect the growth or morphology of this microbe in cell culture^6^. ^Sa^SrtA activity can be detected in cells, but these methods are cumbersome and require antibody detection of sortase-displayed proteins^41^, cell adhesion assays^43^ or incubation of cells with fluorogenic peptidyl sortase substrates that can be slow to label cells^44^.

**Figure 1:**
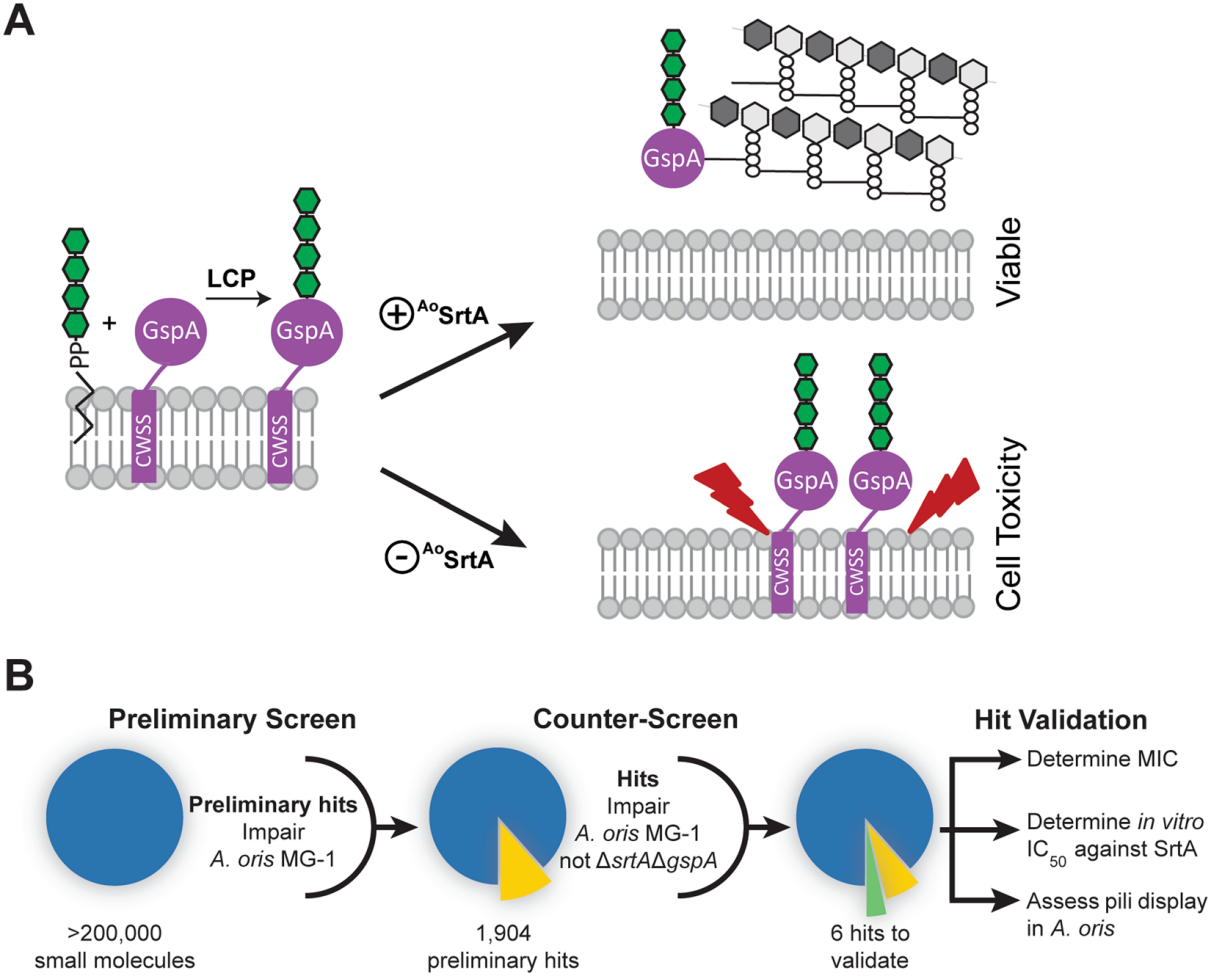
Design and overall work-flow of cell-based inhibitor screen. (**A**) Schematic showing how the activity of the *A*. *oris* SrtA (^Ao^SrtA) enzyme is required for cell viability. A fully functioning ^Ao^SrtA enzyme is needed to attach the glycosylated GspA protein (colored purple with green glycosylation) to the cell wall (top). Reduced gene expression of the ^Ao^SrtA enzyme has been shown to be lethal, presumably because of GspA accumulation in the membrane (bottom). Lethality is dependent upon glycosylation of GspA by the LytR-CpsA-Psr enzymes (LCP). (**B**) Overview of the sortase inhibitor screen. The effects of small molecules on wild-type *A*. *oris* MG-1 was determined for several compound libraries (left). Preliminary hits (1,904) that impaired growth were counter-screened by determining their ability to affect the growth of a Δ*srtA*/Δ*gspA* strain, whose viability is dependent upon the activity of ^Ao^SrtA (middle). Strain-specific growth inhibitors were then validated for sortase inhibitor activity using biochemical and cellular approaches (right). Adobe Illustrator Version: 15.0.0 (https://www.adobe.com/products/illustrator.html).

Recently, Wu *et al*. made the surprising discovery that the viability of the oral bacterium *Actinomyces oris* MG-1 in cell culture depends on the activity of its sortase (^A^°SrtA)^45,46^. ^Ao^SrtA anchors the glycosylated surface protein A (GspA) to the cell wall. In this process, GspA is first glycosylated by the LCP enzyme and then attached to the cell wall by ^Ao^SrtA via lipid II (Fig. 1A, top). Interestingly, reducing ^Ao^SrtA expression causes cell arrest, presumably due to glycol-stress caused by accumulation of glycosylated GspA in the membrane (Fig. 1A, bottom). To the best of our knowledge, *A*. *oris* is the only known bacterium that exhibits a sortase-dependent growth phenotype in cell culture. Here we report the development of a cell-based assay to screen for sortase inhibitors that takes advantage of this unique phenotype. High throughput implementation of the assay was used to screen compound libraries and led to the discovery of several small molecule sortase inhibitors that are validated using biochemical and cellular approaches.

## Results

### Development and implementation of the cell-based screen

Our previous studies have shown that reducing *srtA* gene expression in *A*. *oris* leads to cell death and that this phenotype is conditionally dependent on *gspA* expression^45,46^. To exploit this unique dependence, we developed a cell-based assay to screen for sortase inhibitors that compares the growth-inhibitory effects of small molecules cultured with *A*. *oris* MG-1 (wild-type) versus a Δ*srtA*/Δ*gspA* strain. In the assay, it is presumed that a small molecule sortase inhibitor will selectively impair growth of *A*. *oris* MG-1 by causing GspA to accumulate in the membrane, whereas growth of the Δ*srtA*/Δ*gspA* strain should be unaffected. Prior to implementing the assay in HTS, we optimized conditions for *A*. *oris* growth in 384-well plate format. Both MG-1 and Δ*srtA*/Δ*gspA* strains had similar growth rates and final cell densities in brain-heart infusion broth. Control growth experiments using media containing 1% dimethyl sulfoxide (DMSO) confirmed that both strains are tolerant to this solvent at concentrations used for screening. The growth phenotype of both *A*. *oris* strains was sufficient for use in a HTS, as a Z’ score of 0.81 (MG-1) and 0.71 (Δ*srtA*/Δ*gspA*) was determined when positive (1 µg mL^−1^ penicillin) and negative (media only) control experiments are performed^47^.

The overall workflow for the HTS is shown in Fig. 1B. Initially, small molecules were screened for their ability to kill the wild-type MG-1 strain in 384-well format. Molecules that reduced growth by more than 2.75 standard deviations from the average growth were considered preliminary hits. These molecules were then tested in high-throughput for their ability to impair Δ*srtA*/Δ*gspA* growth, which serves as a counter-screen. Small molecules that exhibited differential growth effects in MG-1 but not Δ*srtA*/Δ*gspA* strains were considered hits and carried forward for further analysis. These hits were validated by determining their minimum inhibitory concentrations (MICs) in cell culture, measuring the *in vitro* half maximal inhibitory concentration of each compound (IC_50_) against the isolated ^Ao^SrtA and ^Sa^SrtA enzymes, and their ability to affect protein display in *A*. *oris* cells.

**Figure 2:**
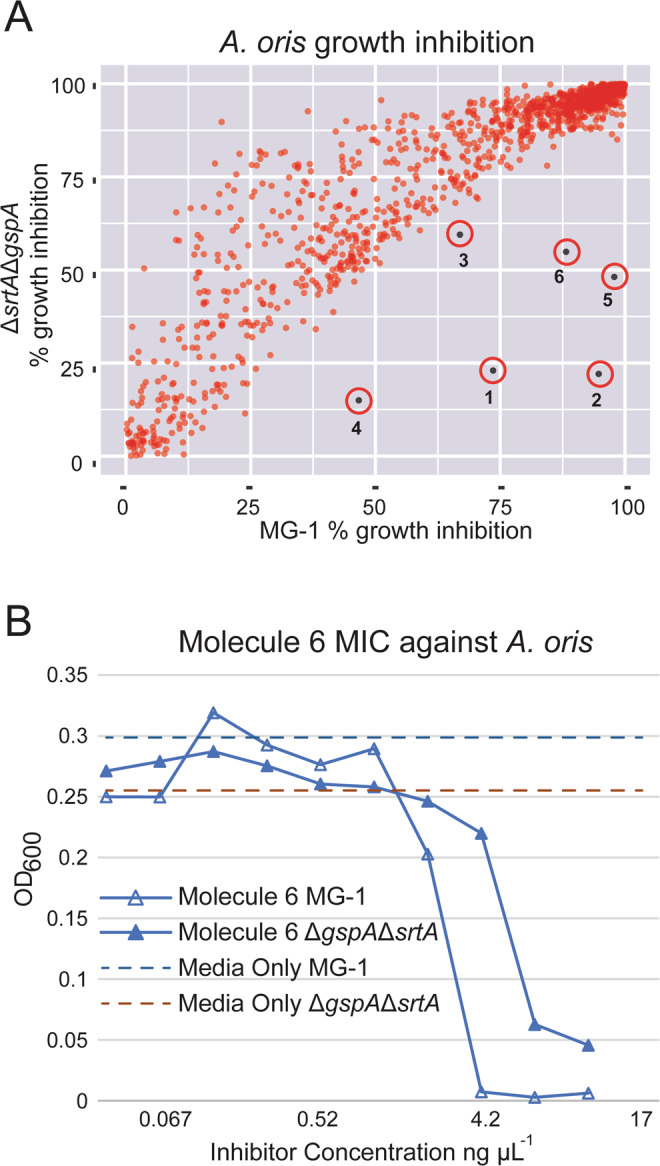
Growth effects of the screened molecules. (**A**) Scatter plot showing the growth effects of small molecules when cultured with either *A*. *oris* wild-type MG-1 or mutant strain Δ*srtA*/Δ*gspA*. The percent growth inhibition for each strain is relative to the parent strain cultured in the absence of the small molecule (Methods). Molecules (**1–6**) exhibited statistically significant strain-specific effects on growth, impairing growth of the wild-type more than the Δ*srtA*/Δ*gspA* strain (circled and labeled). (**B**) Plot showing the effects of molecule 6 on the growth *A*. *oris* MG-1 (open triangles) and Δ*srtA*/Δ*gspA* (filled triangles) strains. The corresponding strains grown in identical conditions in the absence of molecule exhibited OD_600_ values of 0.29 and 0.26, respectively. The data show that **6** has a lower MIC for MG-1 as compared to Δ*srtA*/Δ*gspA*, and is consistent with the HTS data shown in panel (**A**).

In the primary screen, a total of 200,834 small molecules were tested for their ability to impair *A*. *oris* MG-1 growth. Cells were added to an optical density (OD_600_) of 0.01 in media containing 10 µM of each molecule, followed by a 15 hour incubation at 37 °C and end-point OD_600_ measurement. The growth effect of each molecule was normalized and expressed as a percent growth relative to the DMSO-only control (see Methods). A total of 1,904 molecules were classified as preliminary hits (0.95% of the molecules tested), as they reduced MG-1 growth after 15 hours by at least 34%, or 2.75 standard deviations below the average. The preliminary hits were then subjected to a counter-screen in which their growth effects on Δ*srtA*/Δ*gspA* were determined. In order to eliminate potentially erroneously identified preliminary hits, the counter-screen and preliminary screen was performed in duplicate for the 1,904 preliminary hit molecules. A molecule was deemed as a hit if it caused greater than 15% differential growth effect in duplicate when the MG-1 and Δ*srtA*/Δ*gspA* strains were compared. Figure 2 shows a plot of each preliminary hit molecule’s growth effect against the MG-1 and Δ*srtA*/Δ*gspA* strains. Data is plotted as the percentage of growth inhibition for each molecule (see Methods).

**Figure 3:**
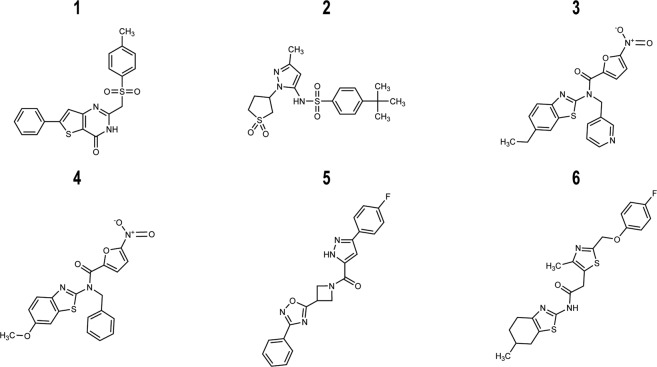
Chemical structures of preliminary hit molecules. Chemical structures of the hit molecules (**1–6**) from the *A*. *oris* screen. These molecules exhibit statistically significant strain-specific effects on growth, impairing the wild-type more than the Δ*srtA*/Δ*gspA* strain. Compounds **3**, **4** and **6** were validated sortase inhibitors, whereas **1**, **2** and **5** selectively impair growth through an unknown, non-sortase dependent mechanism. ChemDraw Professional Version:19.0.1.28 (https://www.perkinelmer.com/product/chemdraw-professional-chemdrawpro).

A total of six compounds preferentially affected MG-1 growth in duplicate, constituting 0.3% of the preliminary hits identified from the primary screen and 0.003% of the total number of compounds tested. The six compounds were termed **1**–**6** and carried forward for further analysis (Fig. 3). R Version: 3.3.3 (https://www.r-project.org/)

### Evaluation of the sortase inhibitor candidates

Two validation assays were performed for each hit molecule that measured: (i) the minimum inhibitory concentration (MIC) required to prevent growth of the MG-1 and Δ*srtA*/Δ*gspA* strains, and (ii) their *in vitro* inhibitory activity against the enzymatic activity of the *A*. *oris* and *S*. *aureus* sortase enzymes. Initially, the selective growth effect originally observed in the HTS that used 384-well plates was more rigorously defined by determining each molecule’s MIC value for the MG-1 and Δ*srtA*/Δ*gspA* strains. Cells were cultured in 100 µL media containing 2-fold dilutions of each small molecule between 320 µM and 0.039 µM (approximately 134 µg mL^−1^ to 0.16 µg mL^−1^). Each of the six preliminary hit molecules (**1–6**) has a lower MIC for the MG-1 strain (between 16.4 µg mL^−1^ (40 µM) and 1.0 µg mL^−1^ (2.6 µM) than for Δ*srtA*/Δ*gspA* (Table 1). Two additional molecules exhibited activity in only one replicate of the high-throughput counter-screen assay; however, we chose to interrogate their activity in proceeding assays to better appreciate the reliability of the HTS. Neither exhibited marked differences in their MIC values when tested in MG-1 and Δ*srtA*/Δ*gspA* strains, which affirms the strength of the duplicate experiment cutoff. Thus, the MIC data confirm the results of the HTS by demonstrating that only the duplicate hit molecules preferentially impair the growth of *A*. *oris* MG-1.

**Table 1:** Growth and inhibitory properties of the preliminary hit molecules.

	Preliminary Hits					
	1	2	3	4	5	6
Differential Growth^a^	65.7 ± 0.5%	96.2 ± 0.3%	17.5 ± 3.5%	38.5 ± 0.5%	96.2 ± 0.3%	71.8 ± 16.5%
MIC (MG-1)^b^	2.0	0.5	8.2	16.4	1.0	2.1
MIC (Δ*gspA/*Δ*srtA*)^b^	4.0	2.1	>130	>130	2.0	8.4
IC_50_ (*A*. *oris*)^c^	>190	>200	30 ± 20	70 ± 50	>190	60 ± 40
IC_50_ (*S*. *aureus*)^c^	80 ± 20	>200	90 ± 20	200 ± 100	110 ± 30	54 ± 8

^a^Differential effects of small molecule on wild-type MG-1 and Δ*gspA*Δ*srtA A*. *oris* strains measured in the high-throughput screen. See methods section complete definition. ^b^Minimum inhibitor concentration (MIC) expressed in μg ml^−1^ for MG-1 and Δ*gspA*Δ*srtA A*. *oris* strains. ^c^Half maximal inhibitory concentration for the *in vitro* enzyme activity of ^Sa^SrtA (*S*. *aureus*) or ^Ao^SrtA (*A*. *oris*) in μg ml^−1^.

The ability of the hit molecules to inhibit sortase activity was measured *in vitro* using an established FRET-based assay^13^ that measures the ability of each enzyme to cleave a fluorogenic peptide substrate; Abz-LAQTG-Dap(Dnp)-NH_2_ and Abz-LPETG-Dap(Dnp)-NH_2_, substrates for ^Ao^SrtA and ^Sa^SrtA, respectively. Each enzyme cleaves between the threonine and glycine bond in these peptides, leading to a measurable increase in fluorescence (see Methods). The IC_50_ value of each compound was determined for both the ^Ao^SrtA and ^Sa^SrtA enzymes. Compounds **3**, **4** and **6** are bona fide enzyme inhibitors, as each inhibits both the ^Ao^SrtA and ^Sa^SrtA enzymes with IC_50_ values ranging from 30–70 µg mL^−1^ (73–170 µM) (Table 1). In contrast, molecules **1**, **2** and **5** failed to inhibit ^Ao^SrtA *in vitro*, suggesting that they selectively affect *A*. *oris* MG-1 growth through a mechanism not involving sortase inhibition.

### Sortase inhibitors affect pilus assembly in *A*. *oris*

**Figure 4:**
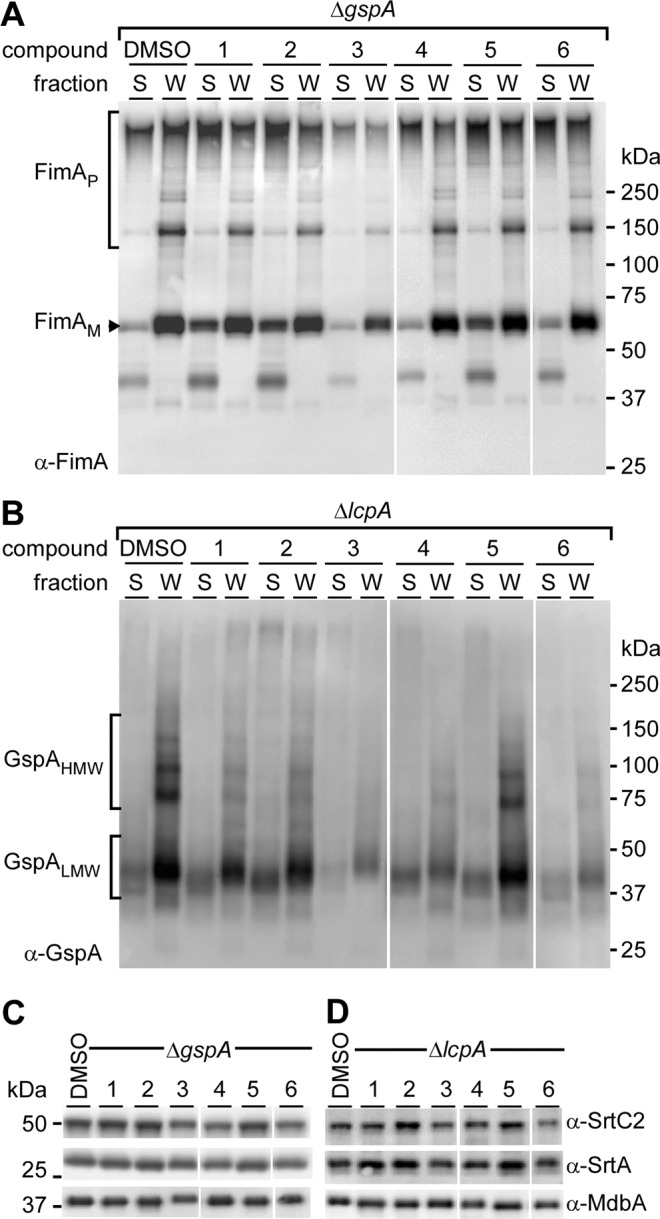
Assessment of cell surface proteins. (**A**,**B**) Cell cultures of *A*. *oris* strains Δ*gspA* (**A**) and *A*. *oris* Δ*lcpA* (**B**) harvested after inhibitor treatment were subjected to cell fractionation. Samples in supernatant culture medium (S) and cell wall (W) fractions were immunoblotted with antibodies against the fimbrial shaft FimA (α-FimA) and glycosylated GspA (α-GspA). (**C**,**D**) The protoplast fractions were subjected to immunoblotting with antibodies against pilus-specific sortase SrtC2 (α-SrtC2), housekeeping sortase SrtA (α-SrtA), and the membrane protein MdbA (α-MdbA) as control.

The MIC and IC_50_ data suggest that at least three of the hit molecules are capable of inhibiting ^Ao^SrtA either within the context of the cell or as an isolated transpeptidase. To understand the physiological effects that these molecules have on the display of pili and surface proteins on the surface of *A*. *oris*, we grew cells with 10 µM of each hit molecule and assessed sortase activity by western blot and electron microscopy as previously reported^45^. To avoid a confounding problem that inhibiting ^Ao^SrtA causes cell arrest, we performed the experiments in a mutant devoid of *gspA*; the aforementioned genetic suppressor of sortase lethality. Consistent with both MIC and IC_50_ data, when samples were immunoblotted with antibodies against the type 2 pilus shaft protein FimA, both molecules **3** and **4** decreased FimA polymers as compared to the control (Fig. 4; compare the first two lanes with lanes containing molecules #3 and #4).

**Figure 5:**
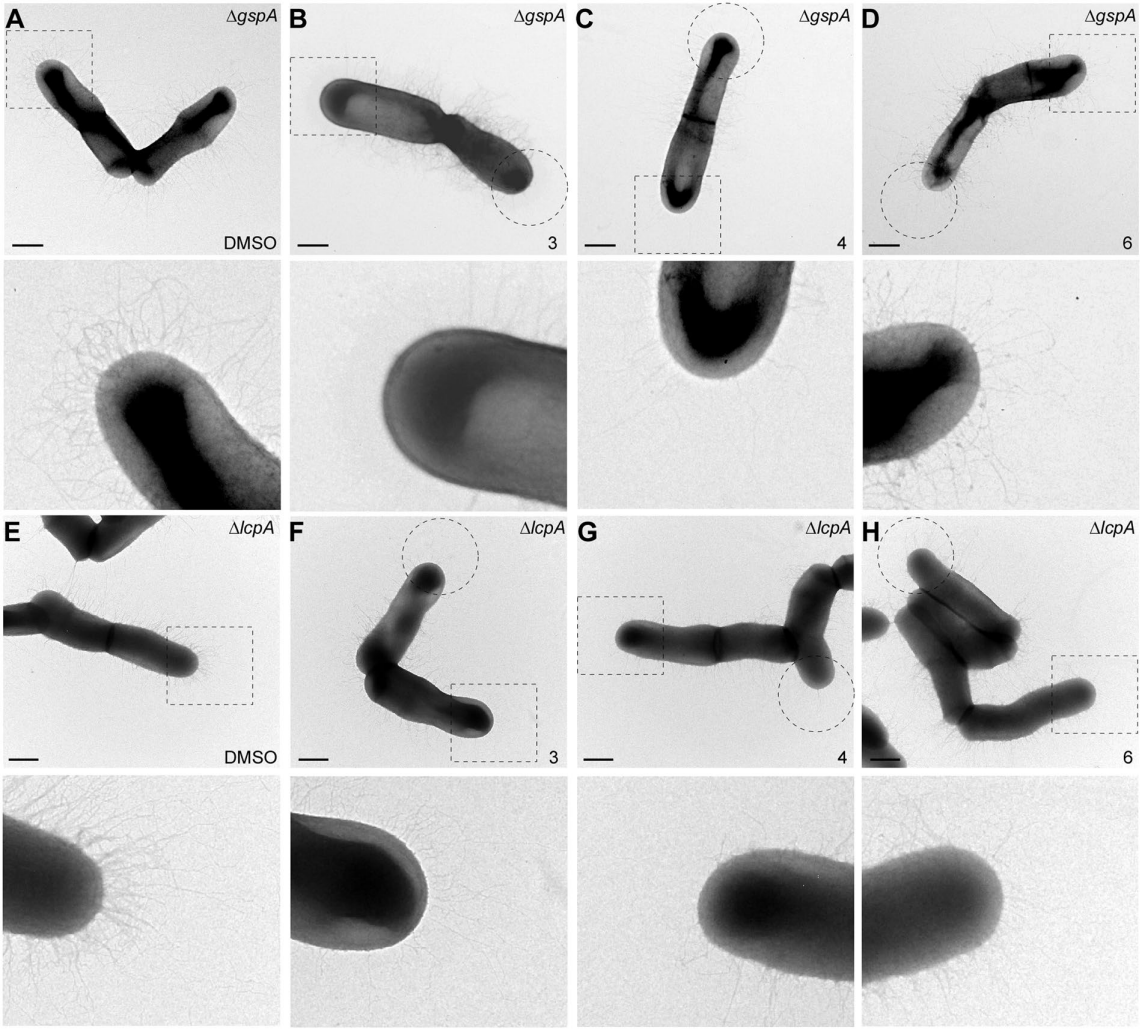
Detection of pili by electron microscopy. Bacterial cultures of strains Δ*gspA* (**A**–**D**) and Δ*lcpA* (**E-H**) treated with compounds **3**, **4** and **6** were subjected to negative staining with 1% uranyl acetate prior to electron microscopy; scale bar of 500 nm. Polar assembly of pili is heighted with dashed circles and rectangles. Enlarged areas in dashed rectangles are shown in below panels.

To examine if these molecules inhibit cell wall anchoring of surface proteins (e.g. GspA), we similarly treated cells devoid of *lcpA*, i.e. Δ*lcpA*, another suppressor of *srtA*^46^. When blotted with antibodies against GspA, no GspA polymers were observed in samples treated with molecules **3**, **4** and **6** (Fig. 4B). To corroborate these observations, we subjected *A*. *oris* cells of Δ*gspA* and Δ*lcp* strains to 10 μM of each molecule (**3**, **4** and **6**) and studied pilus display with transmission electron microscopy (TEM), whereby *A*. *oris* cells were immobilized on nickel grids and stained with 1% uranyl acetate prior to viewing by an electron microscope. Intriguingly, compared to the untreated control, treatment of molecules **3**, **4** and **6** caused significant reduction of pilus assembly at the cell pole in either strain background, i.e. Δ*gspA* or Δ*lcpA* (Fig. 5). The results suggest that these molecules target the nascent pilus assembly machine.

## Discussion

Sortase enzymes are promising drug targets as they mediate the display of important virulence factors in several medically significant bacteria. The *S*. *aureus* ^Sa^SrtA enzyme is particularly interesting because it plays an important role in MRSA infections that are a major cause of human mortality. Despite over fifteen years of effort by a number of research groups, antivirulence agents that work by inhibiting ^Sa^SrtA have yet to be discovered. This may be because nearly all screens for ^Sa^SrtA inhibitors have used the isolated enzyme that is truncated at its N-terminus to increase solubility. Searching for inhibitors using a cell-based assay may yield better results as it could discover small molecules that are capable of traversing the cell wall and targeting the intact enzyme in the microbial membrane. Moreover, it could overcome screening limitations that are caused by the slow enzyme kinetics of the isolated protein. To the best of our knowledge, a robust cell-based sortase assay has yet to be developed, presumably because current methods used to monitor ^Sa^SrtA activity in intact cells are laborious, requiring multi-step immunolabeling and fluorescence-detection experiments. To overcome this limitation, we developed and implemented in high throughput a cell-based assay to identify sortase inhibitors. Our approach exploits the unique growth dependence of *A*. *oris* on the activity of its SrtA enzyme, which is conditionally dependent on glycosylation of the GspA surface protein by an LCP enzyme (Fig. 1A). First, a primary screen was performed in which>200,000 small molecules were tested for their ability to impair the growth of wild-type *A*. *oris* MG-1 (Fig. 1B). Preliminary hit molecules were then counter-screened against the *A*. *oris* Δ*srtA*/Δ*gspA* mutant strain to eliminate small molecules that impaired *A*. *oris* growth via processes unrelated to sortase. In the primary screen, most molecules did not affect *A*. *oris* MG-1 growth when present in the cell culture at 10 μM, as the mean and median percent growth of cultures containing small molecules relative cells grown in standard growth culture containing DMSO was 99.3% and 100.9%, respectively. The interquartile range (IQR) is 12.1%, meaning that 75% of molecules cells grow to 94.2% the average DMSO-only density or better. A total of 1,904 molecules in the screen reduced MG-1 growth to 66% growth (2.75 standard deviations below the average percent growth) and were subsequently counter-screened using the Δ*srtA*/ΔgspA mutant strain. For these molecules, growth effects on each strain were tested in duplicate on separate days to rigorously identify molecules that selectively impaired MG-1 growth. Six preliminary hit molecules were identified, **1–6** (0.32% of the 1,904 MG-1 growth effecting molecules). Each of the six molecules inhibit the growth of the MG-1 strain more than the Δ*gspA*/Δ*srtA* strain when tested in duplicate. The finding that few molecules cause selective growth effects is not surprising, as most molecules can be expected to be generally cytotoxic by inhibiting other essential non-sortase pathways within cell, e.g. division and respiration.

Repeating the counter-screen in duplicate significantly eliminated false positive results. This is evident by our finding that the six preliminary hits also exhibit strain-specific growth effects when they were re-purchased and tested in larger culture volumes (Table 1); each of the preliminary hits (**1–6**) exhibited lower MIC values against MG-1 than against Δ*gspA*/Δ*srtA*. The MIC values against MG-1 fall within a similar range, around 10 μM for all hits, which is expected, as the primary screen and counter-screen monitored growth effects at a single concentration. It is worth noting that the growth screen may have failed to detect potential sortase inhibitors with MIC values significantly greater than 10 μM, meaning higher concentrations of the small molecule are necessary for enzyme inhibition. In principle, this limitation could be overcome by repeating the HTS using higher concentrations of the small molecules in the screen, but this was not performed here because it was cost-prohibitive.

*In vitro* enzyme activity testing reveals that compounds **3**, **4** and **6** inhibit both the ^Ao^SrtA and ^Sa^SrtA enzymes with IC_50_ values ranging from 30 to 70 μg mL^−1^ (73–170 µM). Compounds **3** and **4** are bioisosteres and share related N-(1,3-benzothiazol-2-yl)−5-nitrofuran-2-carboxamide chemical scaffold. In compound **3** the scaffold is elaborated with N-3-pyridylmethyl and 6-ethyl-2-benzothiazole moieties, whereas **4** features benzyl and 5-methoxybenzothiazole. Effectively these compounds are bioisosteres, as the benzene is an isostere with pyridine, as are the ethyl and methoxy groups. Interestingly, molecules containing 5-nitrofuran functional groups are particularly potent at eliciting selective growth effects as they are enriched in our primary hits; 1% of the 1,904 hits from the cell based screen contained this functional group even though it is present in only 0.09% of 200,834 molecules that we screened. Compound **6** is unique, exhibiting a N-methyl-(4,5,6,7-tetrahydro-1,3-benzothiazol-2-yl)-1,3-thiazole-5-carboxamide scaffold. Importantly, compound **6** would not likely have been discovered using conventional FRET-based HTS as it is intrinsically fluorescent and presumably would have been disregarded as a hit. The IC_50_ values for the inhibitors against ^Ao^SrtA are higher than their measured MIC values. This suggests that they may also kill *A*. *oris* cells through non-sortase related mechanisms and/or that they more efficiently inhibit the *A*. *oris* enzyme when it is embedded in the microbial membrane. Preliminary hits **1**, **2** and **5** also effect growth in a strain-specific manner, but do not inhibit ^Ao^SrtA *in vitro* (Table 1). The origins of their selective effects on growth remain to be determined. To the best of our knowledge, none of the hit molecules have been described as antimicrobials, however benzothiazole-based DNA gyrase B inhibitors have been shown to have modest growth inhibitor properties against Gram-positive *Entereoccocus faecalis* when dosed at high concentrations (50 μM)^48^.

Cellular studies indicate that sortase inhibitors **3**, **4** and **6** alter the ability of *A*. *oris* to display GpsA and to assemble pili on its surface. *A*. *oris* uses three distinct sortases to elaborate its cell envelope. ^Ao^SrtA is a class E housekeeping sortase that attaches GspA and other proteins containing the LAQTG sorting signal to the cell wall^49^. ^Ao^SrtA catalyzes a transpeptidation reaction that attaches GspA to lipid II, a cell wall precursor. *A*. *oris* also assembles surface pili (fimbriae) using two class C sortases, SrtC1 and SrtC2. These sortases catalyze transpeptidation by linking protein subunits of the pilus together via lysine-isopepide bonds. Subsequent cell wall anchoring of pilus polymers to peptidoglycan requires ^Ao^SrtA^49^. SrtC1 produces type 1 fimbriae that are comprised of the fimbrial shaft FimP protein and the tip fimbrillin FimQ protein. This structure mediates bacterial adherence to the tooth surface via FimQ interactions with salivary proline-rich protein deposits. The SrtC2 sortase produces type 2 fimbriae, made of the fimbrial shaft FimA protein and tip fimbrillin FimB; these fimbriae are required for bacterial adherence to host cells, biofilm formation, and bacterial coaggregation^50,51^. We performed cell fraction and immunoblot studies to gain insight into how inhibitors **3**, **4** and **6** affect SrtC1 and ^Ao^SrtA sortase activity in intact cells (Fig. 4). Molecule **6** inhibits ^Ao^SrtA in cells as its presence significantly diminishes GspA display (Fig. 4B). In contrast, it has little effect on type 2 pilus production by the SrtC2 sortase as judged by immunoblots of the FimA shaft protein in fractionated cells. The diminished potency of molecule **6** against pili display indicates that it lacks inhibitory activity against class C sortases. This is substantiated by TEM images, which show that molecule **6** only modestly effects pili display in the Δ*gspA* strain (Fig. 5A–D). Since ^Ao^SrtA has been implicated in anchoring of the pilus to the peptidoglycan, one might still expect to see diminished pili display in the absence of the housekeeping sortase; however, it is not uncommon for class C enzymes to compensate for loss of the housekeeping sortase and independently display pili as SrtC2 is capable of catalyzing pilus polymerization and cell wall anchoring of pilus polymers^49,52^. Thus, the data supports molecule **6** as being more selective for ^Ao^SrtA in intact cells.

Cellular studies suggest that compounds **3** and **4** inhibit both class E ^Ao^SrtA and class C SrtC2 sortases. Our interrogation of type 2 pilus assembly reveals that **3** and **4** not only decrease the abundance of pili, but also reduce the amount of higher molecular weight FimA-containing polymers that are being formed (Fig. 4A, compare molecular weight bands ≥150 kDa). Decreased pilin-polymerase activity suggests that SrtC2 is being inhibited. This is substantiated by TEM images of cells treated with these compounds **3** and **4**, which show either completely absent or diminished pili at their poles, respectively (Fig. 5B,C). Inspection reveals that the pili in these cells become gradually shorter toward the poles, suggesting that the SrtC2 enzyme is being inhibited during the 3 hour period of growth. Moreover, cells treated with compounds **3** and **4** have decreased amounts of cell wall associated GspA, compatible with the fact that these molecules also inhibiting the housekeeping ^Ao^SrtA sortase (Fig. 4B). Consistent with the lower IC_50_ against ^Ao^SrtA and ^Sa^SrtA and the lower MIC against MG-1 relative to **4**, we note that **3** appears to more severely limit GspA display than **4** at 10 μM (Figs. 4B and 5B,C**)**. This suggests that **3**, an analog of **4**, is the more potent molecule. It is important to note that these inhibitors were added to actively growing cell cultures that already produced pili. Given the presence of pili at the septal area and significantly reduced pili at the pole (Fig. 5), we surmise that these inhibitors target the new sortase machine at the pole, where the nascent peptidoglycan is synthesized and modified.

In conclusion, we have exploited the unique sortase-dependent growth phenotype of *A*. *oris* to screen small molecule compound libraries for sortase inhibitors. Three molecules, representing two unique scaffolds, inhibit sortase enzymes from both *A*. *oris* and *S*. *aureus* were discovered. This represents the first HTS for sortase inhibitors that relies on the simple metric of cellular growth and suggests that *A*. *oris* is a promising platform for sortase-targeted drug discovery. Future work will need to establish structure-activity relationships for the hit molecules to further increase their potency for potential use as novel anti-infectives, which are urgently needed to treat infections caused by MRSA and other drug-resistant bacterial pathogens.

## Materials and Methods

### Antibiotics, media and consumables

Brain Heart Infusion Broth, modified (BHI) was purchased from Fisher Scientific and prepared as directed within one week of its use. Penicillin G and kanamycin were purchased from Fisher Scientific and stored as directed. 1000x antibiotic stocks were prepared in water, filtered with 0.2 µm syringe filters, and stored at -20 °C until thawed immediately before use. Greiner 384-well plates (EK-30162) and universal lids (EK-2079) were purchased from E&K scientific and used once before disposal as medical waste.

### High-throughput screen

A total of 200,834 small molecule compounds (Molecular Screening Shared Resource, UCLA) were dissolved in 100% Omnisolv methylsulfoxide (MilliporeSigma MX1456P-6) and stored in 384-well polypropylene plates. *Actinomyces oris* MG-1 and Δ*gspA*Δ*srtA* were streaked out on BHI agar plates containing 50 µg/mL kanamycin and grown at 37 °C for 48 hours to allow single colonies to grow. Cultures were started from 2–3 colonies in BHI with 50 µg/mL kanamycin and allowed to grow until an OD_600_ between 0.1–0.4. Meanwhile, 384-well plates were filled with 25 µL BHI broth with 100 µg mL^−1^ kanamycin (columns 1–22) or 200 µg mL^−1^ Penicillin G (positive control, columns 23–24). Five hundred nanoliters of small molecules were transferred into the media (columns 3–22) using the Biomex FX^P^ automated work station with a 384-well pin tool. Precultures were diluted to an OD_600_ of 0.02 in 1 L BHI broth without antibiotics. Twenty five microliters of cell culture was immediately aliquoted into plates containing 25 µL media, antibiotics, and small-molecules using a Multidrop (Thermo LabSystems), resulting in 50 µg mL^−1^ kanamycin and 10 µM small molecules with 1% DMSO or 100 µg mL^−1^ Penicillin G. Plates were immediately lidded and placed into a humidified Cytomat 6001 incubator at 37 °C and allowed to grow for 15.5 hours. Following incubation, plates were removed from the Cytomat using a Thermo Spinnaker robotic arm on a rail, de-lidded, and placed into an EnVision high-speed plate reader for optical density measurement at 620 nm.

Raw data obtained from the screen was formatted in-house to upload to the Collaborative Drug Discovery Vault (www.collaborativedrug.com). Individual optical density readings were converted to a percent growth value ([OD_sample_-OD_Positive control_]/[OD_Negative Control Average_- OD_Positive control_]) and percent growth inhibition (1-percent growth). The Z-factors for the individual molecules were determined. Molecules with a Z-factor less than or equal to -2.75 were considered preliminary hits to advance forward a generous collection of preliminary hit molecules. Five microliters of each of the 1904 preliminary hit molecules were re-arrayed into new 384-well polypropylene plates. Each of the 1904 preliminary hits were assayed against both the MG-1 and Δ*gspA*Δ*srtA* strains in duplicate, on two separate days. Percent difference of growth was calculated according to the equation 100–100[(OD_MG-1, Sample_-OD_MG-1, Positive Control_)/OD_Δ*gspA*Δ*srtA*, Sample_-OD_Δ*gspA*Δ*srtA*, Positive Control_)]/[(OD_MG-1, Average_-OD_MG-1, Positive Control_)/OD_Δ*gspA*Δ*srtA*, Average_-OD_Δ*gspA*Δ*srtA*, Positive Control_)]. A score of 100 indicates that a molecule causes extreme difference in cell survivability between the two strains whereas a score of 0 indicates no differential effect on cellular growth. Molecules were ranked according to their relative growth.

### Hit validation assays

Minimum inhibitory concentration (MIC) values were determined for molecules **1**–**6** according to the Clinical Laboratory Standard Institutes’ (CLSI) Methods for Dilution Antimicrobial Susceptibility Tests for Bacteria that Grow Aerobically, Approved standards-ninth edition; M07-A9 Vol. 32 No. 2 with the following alterations. Overnight cultures of MG-1 and Δ*gspA*Δ*srtA* were diluted to OD_600_ values of 0.01 before being added to BHI containing the appropriate molecule in 96-well plates. Mueller-Hinton broth was not usable because *A*. *oris* is a fastidious bacterium. Plates were sealed with Breatheasy seals and incubated for 18 hours, as *A*. *oris* has a doubling time of 2 hours. Plate seals were removed and OD_600_ values measured. Each plate was run with a positive control (cells, media, and Penicillin G), negative control (media and cells only), and contamination control (media only).

IC_50_ values for the six hit molecules against the ^Ao^SrtA and ^Sa^SrtA enzymes were determined as previously described, with some modifications. Briefly, molecules **1**–**6** were serial diluted 2-fold (from 1.25 mM to 2.44 µM, 500 µM to 0.98 µM final assay concentration) into 25 µM ^Ao^SrtA or ^Sa^SrtA (final assay concentration 10 µM) in buffer A (20 mM HEPES pH 7, 5 mM CaCl_2_, 0.05% TWEEN, 30% DMSO). The final DMSO concentration was 18% in the assay. Samples were incubated 1 hour at room temperature and aliquoted into a 384-well plate (EK-30892). Thirty microliters of FRET peptide (Peptide 2.0) in buffer B (20 mM HEPES pH 7, 5 mM CaCl_2_, 0.05% TWEEN), Abz-LPATG-Dap(Dnp)-NH_2_ for ^Sa^SrtA or Abz-LAQTG-Dap(Dnp)-NH_2_ for ^Ao^SrtA was added and the plate was immediately placed into a Flexstation II plate reader (Molecular Devices) and fluorescence was recorded at 335/420 nm excitation/emission wavelengths after 5 seconds of plate shaking to mix the reaction components. Plate fluorescence was measured over the course of 30 minutes.

### Cell fractionation and western blotting

Cell fractionation and Western blotting were followed according to a published protocol^53^. Briefly, the cultures of *A*. *oris* strains grown in BHI at 37 °C until OD_600_ of 0.25 were aliquoted, and bacterial aliquots were treated with 10 µM of individual sortase inhibitors for 3 hours. Bacterial cultures were then normalized to equal OD_600_, and cells were fractionated into culture medium (S), cell wall (W), and protoplast fractions. Isolated fractions were subjected to protein precipitation by 7.5% trichloroacetic acid, followed by washing with cold acetone, except for the protoplast fractions. Protein samples were dissolved in hot sodium dodecyl sulfate (SDS)-containing sample buffer, separated by 3–12% Tris-glycine gradient gels, and subjected to immunoblotting with specific antisera (α-FimA, 1:10,000 dilution; α-GspA, α-MdbA, α-SrtC2 and α-SrtA, 1:4000 dilution), followed by chemo-luminescence detection.

### Transmission electron microscopy

To observe cell morphology by negative staining, *A*. *oris* cells harvested after inhibitor treatment were washed once with PBS and suspended in 0.1 M NaCl. A drop of 7 µL of bacterial suspension was placed onto carbon-coated nickel grids and stained with 1% uranyl acetate. Samples were examined using a JEOL JEM1200.

## Supplementary information

Supplementary Information.

## Data Availability

All data from this study are available from the corresponding author.
