# Supplementary material for: A Cell-based Screen in Actinomyces oris to Identify Sortase Inhibitors
Source: Sci Rep. 2020 May 22;10:8520. doi: 10.1038/s41598-020-65256-x (PMC7244523; doi:10.1038/s41598-020-65256-x)

**A Cell-based Screen in *Actinomyces oris* to Identify Sortase Inhibitors**

Jason E. Gosschalk^a-b^, Chungyu Chang^c^, Christopher K. Sue^a-b^, Sara D. Siegel^d^, Chenggang Wu^d^, Michele D. Kattke^a-b^, Sung Wook Yi^a^, Robert Damoiseaux^e,f^, Michael E. Jung^a,g^, Hung Ton-That^c,e,^*****, Robert T. Clubb^a-b,g,^*****

^a^ Department of Chemistry and Biochemistry, University of California, Los Angeles;

^b^ UCLA-DOE Institute of Genomics and Proteomics, University of California, Los Angeles;

^c^ Division of Oral Biology and Medicine, University of California, Los Angeles;

^d^ Department of Microbiology and Molecular Genetics, University of Texas Health Science Center, Houston, TX;

^e^ Department of Molecular and Medicinal Pharmacology, University of California, Los Angeles;

^f^ California NanoSystems Institute, University of California, Los Angeles;

^g^ Molecular Biology Institute, University of California, Los Angeles, 611 Charles Young Drive East, Los Angeles, CA 90095, USA.

*****To whom correspondence should be addressed:

Robert T. Clubb

Department of Chemistry and Biochemistry, University of California, Los Angeles, 611 Charles Young Drive East, Los Angeles, CA 90095, USA

Tel. (+1) 310 206 2334; Email: [rclubb@mbi.ucla.edu](mailto:rclubb@mbi.ucla.edu)

Hung Ton-That

Division of Oral Biology and Medicine, School of Dentistry, University of California, Los Angeles, CA 90095, USA

Tel. (+1) 310 267 5910; Email: htonthat@dentistry.ucla.edu

**Supplementary Information**

Supplementary Figure S1. Full Length Gels for Figure 4.

Presented are the original, uncropped images used to generate Figure 4 presented in the main text.


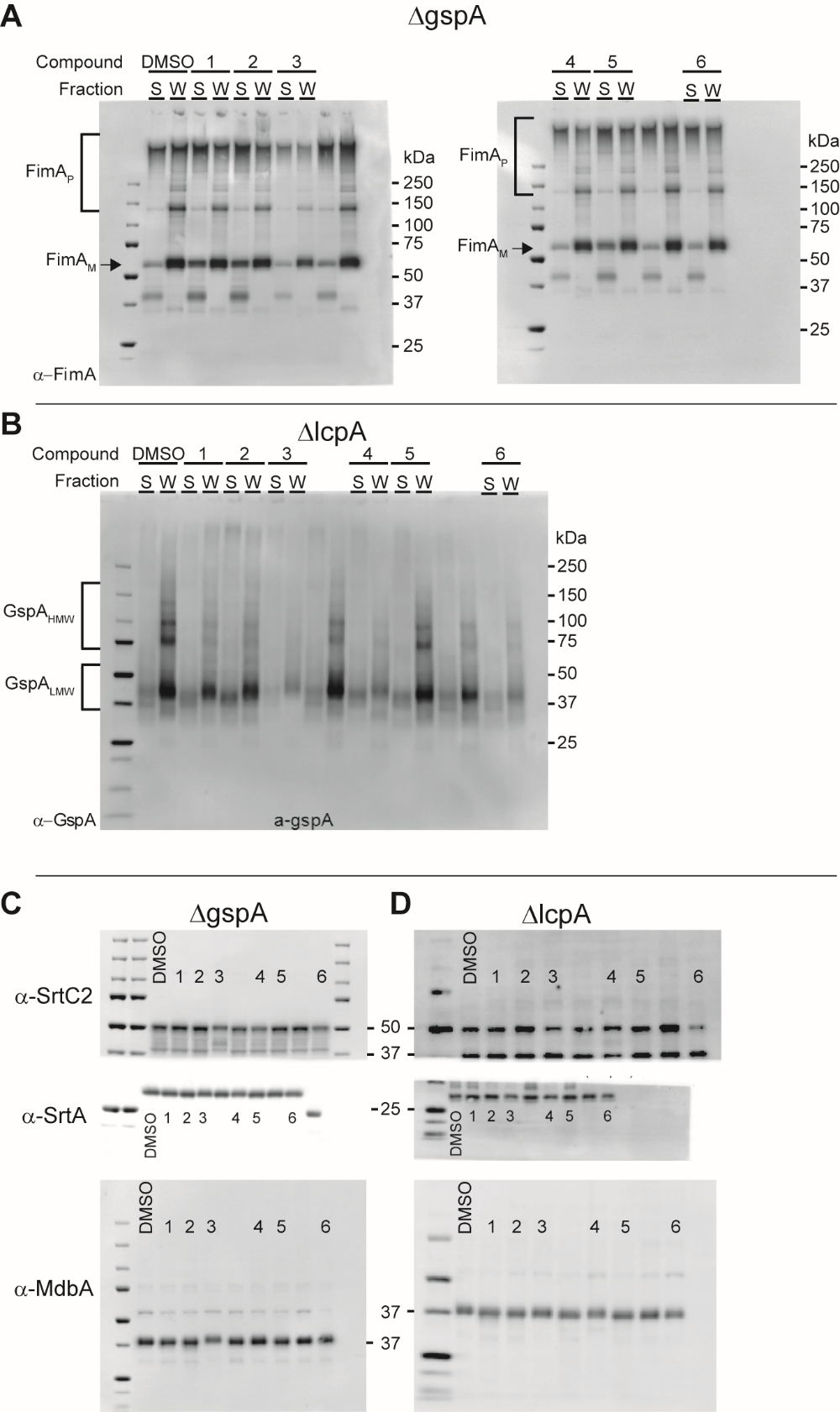

Supplement: Supplementary file 1 — Supplementary Information. [file 41598_2020_65256_MOESM1_ESM.docx]
